# The differential expressions of 78-kDa glucose-regulated protein of infiltrating plasma cells in peripheral joints with the histopathological variants of rheumatoid synovitis

**DOI:** 10.1186/ar2588

**Published:** 2009-01-09

**Authors:** Weijia Dong, Xiaoyan Li, Yuan Feng, Chunmei Fan, Zhinan Chen, Ping Zhu

**Affiliations:** 1 Department of Clinical Immunology, State Key Discipline of Cell Biology, Xijing Hospital, Fourth Military Medical University, 17 Changlexi Street, Xi'an 710032, Shaanxi, PR China; 2 Cell Engineering Research Center, State Key Discipline of Cell Biology, Fourth Military Medical University, 17 Changlexi Street, Xi'an 710032, Shaanxi, PR China

## Abstract

**Introduction:**

The local production of pathogenic autoantibodies by plasma cells in synovium is one of the hallmarks of rheumatoid arthritis (RA). There may be a potential link between ectopic lymphoid neogenesis and the local autoimmunity in rheumatoid synovium. The unfolded protein response (UPR) has key roles in the development and maintenance of plasma cells secreting immunoglobulin. This study was designed to explore the potential links between the activation of the UPR of infiltrating plasma cells in inflamed peripheral joints and the histopathological variants of rheumatoid synovitis as well as the local production of pathogenic autoantibodies.

**Methods:**

The variants of rheumatoid synovium were histopathologically classified into follicular and diffuse synovitis. Immunohistochemical and double-immunofluorescent stainings were performed to detect the expression of 78-kDa glucose-regulated protein (GRP78), a marker of activation of the UPR, in infiltrating plasma cells of synovium, and flow cytometry and immunoblotting analyses were performed to quantify GRP78 in plasma cells of synovial fluid in inflamed peripheral joints of RA. The detections were also taken in osteoarthritis (OA) as controls. The synovial fluid levels of anti-cyclic citrullinated peptide antibodies (anti-CCP) (IgG) were quantified with the enzyme-linked immunosorbent assay and corrected to those of total IgG in RA.

**Results:**

Expressions of GRP78 were more intensive in infiltrating plasma cells in RA synovium relative to those in OA synovium (*P *< 0.001) and in synovium with follicular synovitis relative to that with diffuse synovitis (*P *< 0.001). Analyses by flow cytometry and immunoblotting showed that there was a significant upregulation of GRP78 of plasma cells from synovial fluid of RA compared with that of OA (*P *< 0.05) and from synovial fluid of follicular synovitis relative to that of diffuse synovitis (*P *< 0.05). Moreover, a positive relationship between the expression of GRP78 of plasma cells from synovial fluid and the corrected synovial levels of anti-CCP (IgG) was seen in RA (*P *< 0.001).

**Conclusions:**

There may be a link between enhanced activation of the UPR of plasma cells and ectopic lymphoid neogenesis as well as the local production of anti-CCP (IgG) in inflamed peripheral joints of RA.

## Introduction

Rheumatoid arthritis (RA) is a systemic inflammation disease characterized by chronic and invasive synovitis that causes cartilage destruction and subchondral bone erosion [1]. The infiltrating plasma cells in rheumatoid synovium could synthesize the pathogenic autoantibodies such as anti-cyclic citrullinated peptide antibodies (anti-CCP) [2], which can be of both diagnostic and prognostic value for early-onset or established RA [3,4]. In addition, it has previously been documented that there may be a potential link between ectopic lymphoid neogenesis, which is characterized by the formation of lymphoid follicle with germinal center response and can facilitate the terminal differentiation of B cells into plasma cells in rheumatoid synovium [5], and the local production of high-affinity pathogenic autoantibodies [6,7].

In rheumatoid synovium, the terminal differentiation of B cells into plasma cells in response to antigenic stimuli could require a massive increase in the biosynthetic capacity to produce the autoantibodies within the endoplasmic reticulum (ER) [8-10]. The ER stress response or activation of the unfolded protein response (UPR) can ensue. The UPR can play key roles in the development and maintenance of the plasma cells secreting immunoglobulin [8,9] and may be essential to allow plasma cells to become secretary factories dedicated to high-level autoantibody production [11,12].

Activation of the UPR in plasma cells can promote the expression of ER chaperones, such as 78-kDa glucose-regulated protein (GRP78), mainly via ER transmembrane protein Ire1 (inositol-requiring kinase 1) and ATF6 (activating transcription factor 6) signaling pathways [10,11,13]. GRP78, which is also referred to as immunoglobulin heavy-chain-binding protein (BiP), is a molecular chaperone that binds transiently to proteins traversing through the ER and facilitates their folding, assembly, and transport. As the master regulator of the ER, GRP78 represents an important prosurvival component of the secretary cells, including antibody-secreting plasma cells [14-16]. Moreover, the induction of GRP78 can be used for the quantitative measurement of events in activation of the UPR [16].

Previous studies have indicated that GRP78/BiP is overproduced in inflamed synovium [17] and may have immunogenic roles in driving the local and systemic autoimmunity in RA [18,19]. In addition, GRP78/BiP has been reported to exert regulatory activities for inflammation and to prevent the inflammatory lesions in experimental arthritis [18]. Nevertheless, there were few reports on the expression of GRP78/BiP or its potential link with the histopathological variants of rheumatoid synovitis and the local production of autoantibodies or on the induction of the UPR in infiltrating plasma cells within rheumatoid peripheral joints. In the present work, we investigated the expression of GRP78 of plasma cells in both synovial tissue and fluid in inflamed peripheral joints of RA, trying to explore the expressional profiles of GRP78 of plasma cells in distinct histological variants of rheumatoid synovitis and thus to determine whether there was a potential link between activation of the UPR of plasma cells and ectopic lymphoid neogenesis in rheumatoid synovium as well as the local production of pathogenic autoantibody such as anti-CCP.

## Materials and methods

### Patients and samples

Synovium and synovial fluid were taken at total knee arthroplasty or arthroscopic synovectomy for the inflammatory peripheral joints in Xijing Hospital from 24 RA patients (7 males and 17 females). All of the RA patients fulfilled the 1987 revised diagnostic criteria of the American College of Rheumatology [20]. The mean age of the patients was 40.6 ± 11.9 years, and the median disease duration was 3.0 years. The mean erythrocyte sedimentation rate was 47.1 ± 20.4 mm/hour, and the mean serum level of C-reactive protein was 3.85 ± 2.04 mg/dL. Among all of the 24 RA patients, 18 were seropositive and 6 were seronegative for anti-CCP (IgG) (cutoff value of 5 RU/mL). Synovium tissues from 12 osteoarthritis (OA) patients and synovial fluid from 10 of 12 OA patients were also obtained under diagnostic arthroscopy as controls. Ethics approval for this study was granted from the medical ethics committee of Xijing Hospital, and all of the subjects gave their informed consent.

### Histopathological evaluations

Synovial tissue samples underwent routine staining with hematoxylin and eosin (HE). The variants of rheumatoid synovitis were analyzed under light microscopy (BX60; Olympus, Tokyo, Japan) and determined mainly as previously described [6,21], with particular attention to cell infiltrating density, the topographical arrangement of lymphocytes and macrophages, the distribution of high endothelial venules, and the relationship of lymphocytes to the adjacent vasculars. The variants of rheumatoid synovitis in this study were subsequently categorized into follicular synovitis characterized by the formation of discrete lymphoid follicles, part of which presented the germinal center reaction with apparent central clearing of the lymphoid aggregates, and into diffuse synovitis characterized by diffuse lymphocyte infiltrations in the sublining layers of rheumatoid synovium without further microanatomical organization.

### Immunohistochemical staining

The streptavidin/peroxidase (SP) immunohistochemical stainings were performed in synovium from all of the 24 RA and 12 OA patients. Briefly, serial synovium sections (4 to 5 μm thick) embedded in paraffin were dewaxed and hydrated. The sections were placed in 3% H_2_O_2 _in methanol to block endogenous peroxidase, incubated with 10% normal rabbit nonimmune serum for 10 minutes to minimize background staining, and incubated with 1:100 anti-GRP78 goat polyclonal antibody (Santa Cruz Biotechnology, Inc., Santa Cruz, CA, USA) for 60 minutes at room temperature. Normal goat serum IgG (Santa Cruz Biotechnology, Inc.) was used as a control for the primary antibody. After a wash with phosphate-buffered saline (PBS) (pH 7.2 to 7.4), the sections were incubated with biotinylated rabbit anti-goat secondary antibody (Zymed Laboratories, Inc., now part of Invitrogen Corporation, Carlsbad, CA, USA) for 10 minutes at room temperature. The sections were then incubated with peroxidase-conjugated streptavidin (Invitrogen Corporation) for 10 minutes. After a wash with PBS, substrate reagents (diaminobenzidine) were added. Finally, the counterstaining was performed with hematoxylin and the sections were mounted.

To facilitate statistical analysis, GRP78 immunoperoxidase staining for infiltrating plasma cells of two representative sections per patient was scored by three researchers (XL, YF, and CF), and the mean values were reported as follows: 1 = no or weak staining, 2 = moderate staining, 3 = strong staining, and 4 = very strong staining. Researchers were not informed of the data of the patients. When the GRP78 staining of infiltrating plasma cells of synovium from the patients was not scored consistently, discussions were performed until agreements were reached among the three researchers.

### Double-immunofluorescent staining

Fresh synovial tissue specimens from all of the RA and OA patients were snap-frozen immediately, and serial cryostat sections (6 μm thick) were prepared. After being fixed with 4% paraformaldehyde, the sections were treated with 10% normal rabbit nonimmune serum for 30 minutes and then incubated with 1:100 anti-CD138 mouse monoclonal antibody (Serotec, Oxford, UK) and 1:100 anti-GRP78 goat polyclonal antibody (Santa Cruz Biotechnology, Inc.) overnight at 4°C. The normal mouse and goat IgGs (Santa Cruz Biotechnology, Inc.) were used to replace the primary antibodies as negative controls. After a wash in PBS (pH 7.2 to 7.4), the sections were incubated with 1:100 cyanine-3-labeled sheep anti-mouse IgG (Sigma-Aldrich, St. Louis, MO, USA) and 1:100 fluorescein isothiocyanate (FITC)-labeled rabbit anti-goat IgG (Sigma-Aldrich) in PBS (containing 1% bovine serum albumin [BSA], pH 7.2 to 7.4) in the dark for 1 hour. Finally, the sections were washed with PBS and mounted with glycerol. The sections were then analyzed and photographed with a confocal laser scanning microscope (FV 1000; Olympus).

### Flow cytometry analysis

Mononuclear cells from heparinized synovial fluid of 24 RA and 10 OA patients were incubated with hyalidase (Sigma-Aldrich) at 37°C for 30 minutes before being isolated with the Ficoll-Hypaque (Sigma-Aldrich) gradient centrifugation method. Separated synovial fluid cells (10^6^/mL) were incubated with phycoerythrin-conjugated anti-CD138 mouse monoclonal antibody (BD Biosciences, San Jose, CA, USA) and FITC-conjugated anti-GRP78 goat polyclonal antibody (Santa Cruz Biotechnology, Inc.). The FITC-conjugated goat IgG (SouthernBiotech, Birmingham, AL, USA) was used as a control. Before being incubated with FITC-conjugated anti-GRP78 goat polyclonal antibody, the synovial cells were treated with fluorescence-activated cell sorting (FACS) permeabilizing solution (BD Biosciences). CD138^+ ^cells were gated on lymphocytes of synovial fluids, and 5,000 events were measured. Cells were analyzed with FACS Calibur flow cytometry (BD Biosciences). Data were processed using Cell Quest software (BD Biosciences).

### Plasma cell isolation and Western blotting

Mononuclear cells isolated from synovial fluid of the 24 RA and 10 OA patients, as mentioned above, were sorted by CD138 microbeads (Miltenyi Biotec, Auburn, CA, USA) in accordance with the manufacturer's instructions. Briefly, mononuclear cells were incubated at a concentration of 1 × 10^8 ^cells per milliliter with CD138 microbeads at a titre of 1:5 for 30 minutes at 4°C. Then the cells were washed once with PBS buffer containing 5 mM EDTA (ethylenediaminetetraacetic acid) and 0.5% BSA (PBS/EDTA/BSA). After being resuspending in 2 mL of PBS/EDTA/BSA, cells were separated using two sequential MS columns (Miltenyi Biotec). After estimation of the percentage of plasma cells expressing CD138 by FACS analysis (>95%), the isolated cells were lysed at 4°C for 1 hour in the lysis buffer, consisting of 20 mM Tris (pH 7.8), 100 mM NaCl, 10 mM EDTA, 1% Triton X-100, 5 mM iodoacetamide, 5 μg/mL aprotinin, 10 μg/mL leupeptin, 10 μg/mL pepstatin A, 0.04% sodium azide, and 1 mM phenylmethylsulphonyl fluoride. Nuclei and cell debris were then pelleted at 10,000 *g *for 5 minutes at 4°C, and the supernatants were used for Western blotting.

Supernatant protein concentrations were firstly assessed with a bicinchoninic acid protein assay reagent kit (Pierce Biotechnology, now part of Thermo Fisher Scientific Inc., Rockford, IL, USA) in accordance with the manufacturer's protocols, and the plasma cell extracts were finally adjusted to 30 μg of total protein per sample in Laemmli buffer. Then the supernatants were electrophoresed in SDS-PAGE according to the method of Laemmli [22]. After electrophoresis, the gels were blotted onto nitrocellulose filter membranes (Bio-Rad Laboratories, Hercules, CA, USA). After blocking with 4% defatted milk powder in TBST (1 mL of Tween 20 in 1 L of Tris-buffered saline) at room temperature for 1.5 hours, the membranes were incubated with a 1:1,000 dilution of anti-GRP78 rabbit polyclonal antibody (Santa Cruz Biotechnology, Inc.) or 1:500 anti-actin rabbit antibody (Sigma-Aldrich) for 1 hour. Anti-GRP78 rabbit polyclonal antibodies were omitted as conjugate controls. The membranes were then incubated with 1:3,000 horseradish peroxidase-labeled goat anti-rabbit IgG (Santa Cruz Biotechnology, Inc.) at room temperature for 1 hour. Between the above steps, the membranes were washed with TBST for 5 minutes × 3. Finally, the immunolabeled bands were visualized by chemiluminescence with an enhanced chemiluminescence Western blot detection kit (Amersham Biosciences, now part of GE Healthcare, Little Chalfont, Buckinghamshire, UK), exposed to film, and quantified with the GeneGnome and GeneTools image scanning and analysis package (Syngene Ltd., a division of the Synoptics Group, Cambridge, UK). Prestained standards of low-range molecular weight were used to determine protein size (Sigma-Aldrich). The raw values of densitometric analyses were used to identify the expressional intensities of GRP78 and actin. The relative levels of expression of GRP78 of plasma cells were determined by the expressional intensities being normalized to actin.

### Synovial fluid levels of anti-CCP

Detection of anti-CCP (IgG) of the synovial fluids of RA joints was performed with an enzyme-linked immunosorbent assay kit (Euroimmun, Lübeck, Germany) in accordance with the manufacturer's protocols. Patient samples were diluted 1:101 in sample buffer. According to the standard curve made from the five calibration sera, the synovial fluid levels of anti-CCP (RU/mL) were quantified. If the extinction value of a patient sample lay above that of calibrator 5 (200 RU/mL), the sample was measured in a new test run at a dilution of 1:400 and the result in relative units per milliliter read from the standard curve for this sample was then multiplied by a factor of 4. Finally, the corrected synovial fluid levels of anti-CCP were determined by being normalized to those of total IgG (g/L), which were determined by the reagents kit of the Immage Immunochemistry System (Beckman Coulter, Inc., Fullerton, CA, USA).

### Statistics

Data are expressed as mean ± standard deviation. Statistical analysis was performed with the Mann-Whitney *U *test in SPSS 12.0 for Windows (SPSS Inc., Chicago, IL, USA) for comparison of the means and with the Spearman's rho for the correlation tests. *P *values of less than 0.05 were considered statistically significant.

## Results

### Histopathological patterns of rheumatoid synovium

**Figure 1:**
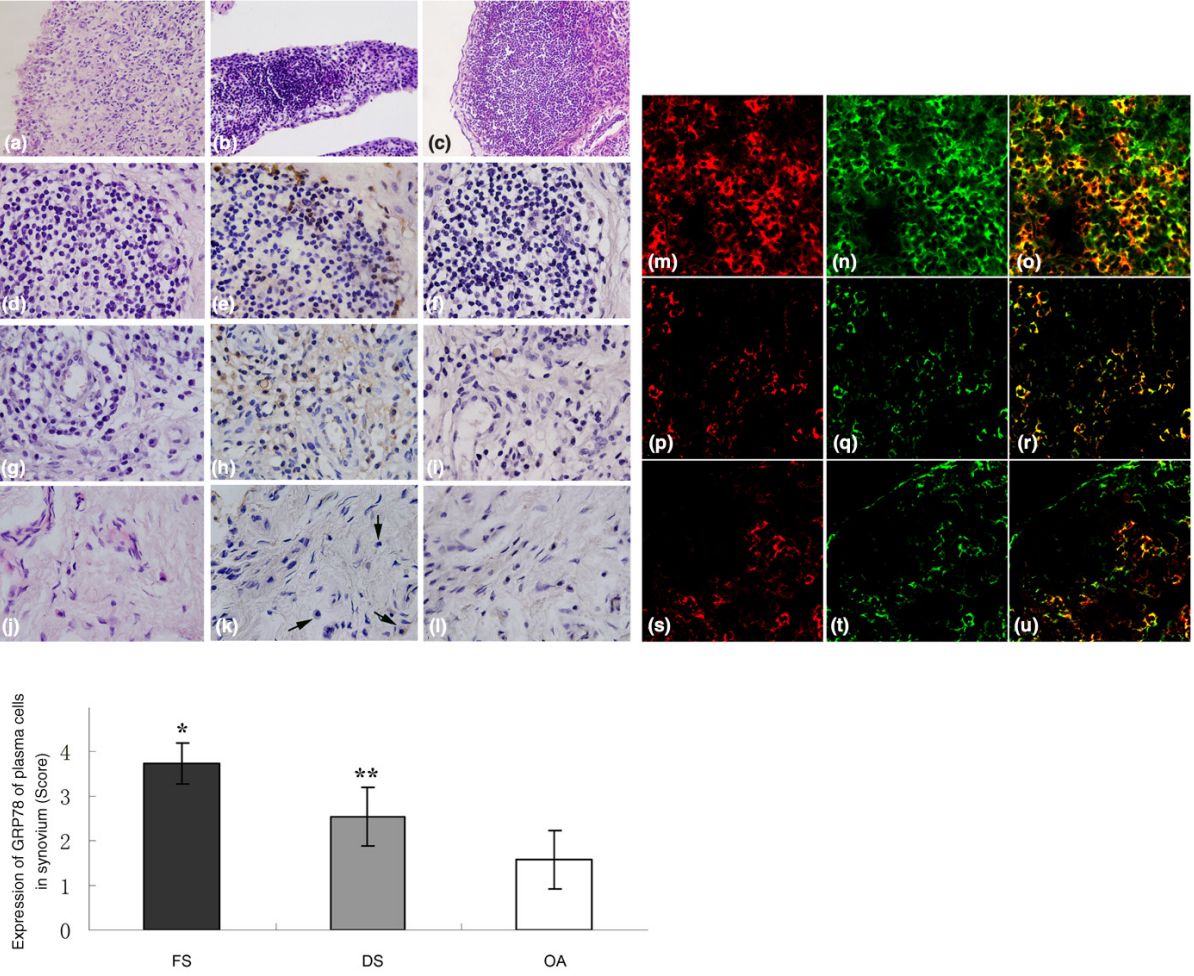
**Histopathological patterns and 78-kDa glucose-regulated protein (GRP78) staining of rheumatoid synovium**. The typical synovium with diffuse synovitis is shown in **(a)**. Synovium presented diffuse lymphocyte infiltration without specific microanatomical organization. The representative rheumatoid synovium with follicular synovitis is shown in **(b)**. The lymphoid aggregates are extensive and assume the appearance of discrete lymphoid follicles with germinal center formation. The reactive germinal center, characterized by pale-staining centroblasts with deeply stained mantle zones, is shown in **(c)**. Representative immunohistochemical stainings for the sequential sections of follicular synovitis **(d-f)**, diffuse synovitis **(g-i)**, and osteoarthritis (OA) synovium **(j-l) **are presented. Frames (d), (g), and (j) show hematoxylin and eosin stainings. In follicular synovitis, GRP78 stainings for plasma cells surrounding lymphoid follicles are clear and intensive in contrast to no or only weak stainings for small lymphocytes and large centroblasts within lymphoid follicles (e) and moderate GRP78 stainings for infiltrating plasma cells in diffuse synovitis (h). There are no or only weak GRP78 stainings for plasma cells (arrows) in OA synovium (k). The normal goat serum IgG replaces the primary antibody as a negative control (f, i, and l). Representative double-immunofluorescent stainings in synovium from rheumatoid arthritis (RA) and OA patients are shown in **(m-u)**. Frames (m), (p), and (s) show the expression of CD138 (bright red fluorescent light), and frames (n), (q), and (t) show the expression of GRP78 (bright green fluorescent light) in synovium with follicular synovitis, diffuse synovitis, and OA, respectively. Frames (o), (r), and (u) (merged) reveal the coexpression of CD138 and GRP78. The bar graph shows that there is a statistical difference in the expression of GRP78 of infiltrating plasma cells between RA (follicular synovitis + diffuse synovitis) and OA synovium (**P < 0.001) as well as between follicular and diffuse synovitis (*P < 0.001). Original magnifications: ×400 (a-c), ×1,000 (d-l), and ×800 (m-u). DS, diffuse synovitis; FS, follicular synovitis.

Synovia from 13 RA patients analyzed by HE staining showing that there were diffuse lymphocyte infiltrations without further microanatomical organization were classified as diffuse synovitis in this study. In synovia sampled from the other 11 RA patients, lymphoid aggregates were extensive and assumed the appearance of discrete lymphoid follicles, part of which presented the formation of germinal center, and the synovia were categorized as follicular synovitis. Nine of 11 of RA patients with follicular synovitis and 9 of 13 of those with diffuse synovitis were seropositive for anti-CCP. Fibrinoid necrobiotic granulomas were found in synovia from only 2 RA patients with follicular synovitis. Figure 1a–c shows the typical synovia from patients with the variants of rheumatoid synovitis.

### The expression of GRP78 of infiltrating plasma cells in rheumatoid synovium

The infiltrating plasma cells in both RA and OA synovia could be clearly identified by their eccentrically positioned nuclei surrounded by abundant cytoplasm and often a perinuclear halo. The SP immunohistochemical stainings showed the clear and intensive stainings of GRP78 in infiltrating plasma cells in RA synovium, especially in those with follicular synovitis, in contrast to no or only weak stainings in infiltrating plasma cells in OA synovium (Figure 1d–l). The double-immunofluorescent staining showed the localization of GRP78 in membrane surface and cytoplasm of infiltrating plasma cells, which were positive for CD138 staining (Figure 1m–u). The mean expressional scores of GRP78 of infiltrating plasma cells were 3.73 ± 0.47 in synovium with follicular synovitis, 2.54 ± 0.66 in synovium with diffuse synovitis, and 1.58 ± 0.67 in those with OA. The significant differences of GRP78 staining of infiltrating plasma cells between RA and OA synovium and between follicular and diffuse synovitis, as analyzed by the Mann-Whitney *U *test, are shown in the bar graph in Figure 1.

### The expression of GRP78 of plasma cells in rheumatoid arthritis synovial fluid

**Figure 2:**
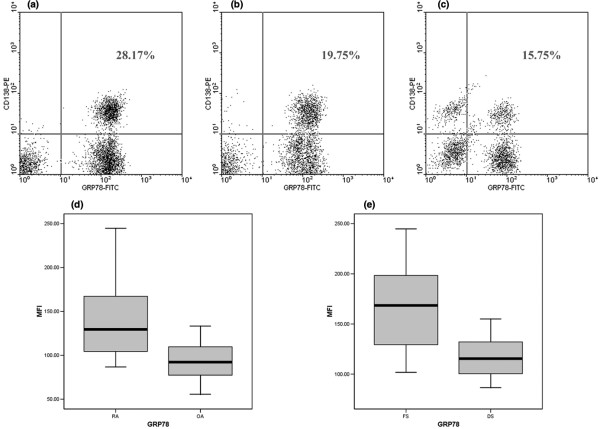
**Flow cytometry analysis of the expression of 78-kDa glucose-regulated protein (GRP78) in plasma cells of synovial fluid**. The representative expressions of GRP78 in CD138^+ ^plasma cells of synovial fluid of follicular synovitis, diffuse synovitis, and osteoarthritis (OA) are shown in **(a-c)**, respectively. The significantly increased mean fluorescent intensity of GRP78 in CD138^+ ^plasma cells of rheumatoid arthritis (RA) relative to OA (*P *= 0.001) and the upregulated mean fluorescent intensity of GRP78 in CD138^+ ^plasma cells of follicular synovitis relative to that of diffuse synovitis (*P *= 0.002) are shown in **(d) **and **(e)**, respectively. DS, diffuse synovitis; FITC, fluorescein isothiocyanate; FS, follicular synovitis; MFI, mean fluorescent intensity; PE, phycoerythrin.

Flow cytometry analyses showed that the mean fluorescent intensities of GRP78 of CD138^+ ^plasma cells of synovial fluid were 141.2 ± 43.6 in RA and 93.1 ± 24.4 in OA. The mean fluorescent intensities of GRP78 of CD138^+ ^plasma cells were 169.8 ± 46.8 in follicular synovitis and 117.1 ± 21.0 in diffuse synovitis. Figure 2 shows that there were significant differences in mean fluorescent intensity of GRP78 of CD138^+ ^plasma cells of synovial fluid between RA and OA as well as between follicular and diffuse synovitis.

**Figure 3:**
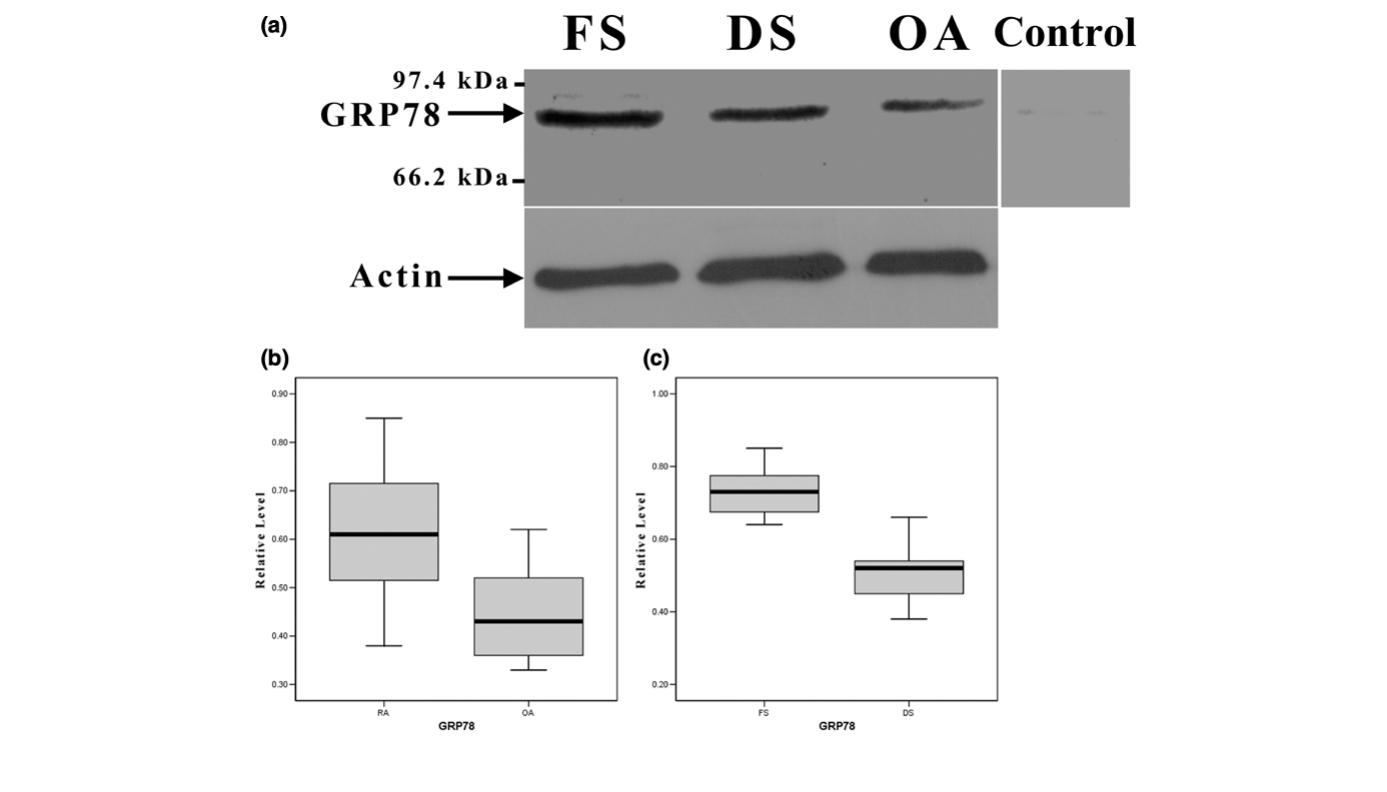
**Western blotting analysis of 78-kDa glucose-regulated protein (GRP78) in isolated plasma cells from synovial fluids**. The representative expressions of GRP78 in plasma cells of follicular synovitis, diffuse synovitis, and osteoarthritis (OA) are shown in **(a)**. The representative conjugate control for detection of GRP78 in plasma cells of follicular synovitis is also shown in (a). The upregulated GRP78 in plasma cells of rheumatoid arthritis (RA) relative to that of OA (*P *= 0.002) and the increased expression of GRP78 in plasma cells of follicular synovitis relative to that of diffuse synovitis (*P *< 0.001) are shown in **(b) **and **(c)**, respectively. DS, diffuse synovitis; FS, follicular synovitis.

Western blotting analyses showed that the mean normalized relative levels of expression of GRP78 of CD138^+ ^plasma cells from synovial fluid were 0.61 ± 0.14 in RA and 0.45 ± 0.10 in OA. The mean relative levels of expression of GRP78 of CD138^+ ^plasma cells were 0.73 ± 0.07 in follicular synovitis and 0.51 ± 0.08 in diffuse synovitis. Figure 3 shows that there was a significant upregulation of GRP78 of synovial plasma cells of RA relative to that of OA and an upregulation of GRP78 of synovial plasma cells of follicular synovitis relative to that of diffuse synovitis.

### Relationship between the expression of GRP78 of plasma cells in synovial fluid and the corrected synovial fluid levels of anti-CCP in inflamed rheumatoid arthritis peripheral joints

**Figure 4:**
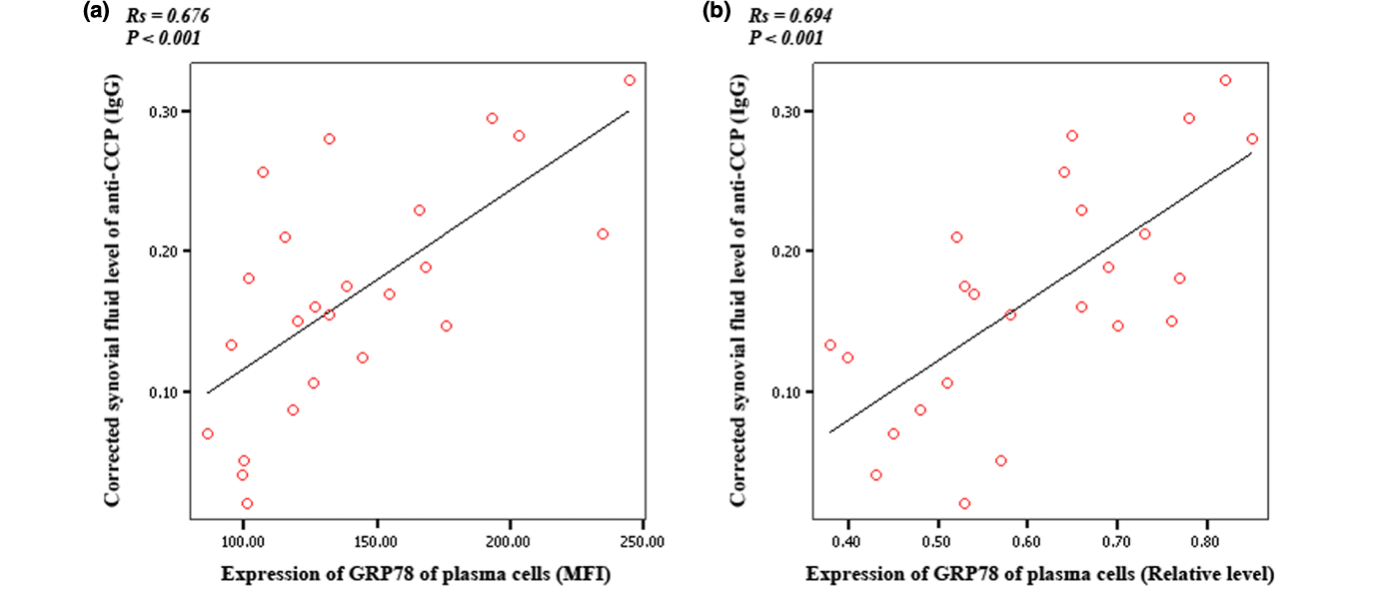
**Relationship between expressions of 78-kDa glucose-regulated protein (GRP78) of synovial plasma cells and corrected synovial fluid level of anti-cyclic citrullinated peptide antibodies (anti-CCP) (IgG) in rheumatoid arthritis**. The highly positive associations of the expression of GRP78 of plasma cells detected by flow cytometry and Western blotting with the corrected synovial fluid level of anti-CCP (× 10^5 ^RU/g per L) are presented in **(a) **(Rs = 0.676, *P *< 0.001) and **(b) **(Rs = 0.694, *P *< 0.001), respectively. MFI, mean fluorescent intensity.

The mean synovial fluid levels of anti-CCP (IgG) and IgG were 113.1 ± 53.9 RU/mL and 709.8 ± 304.8 mg/dL, respectively, in all of the RA patients. The mean corrected synovial fluid level of anti-CCP (IgG) was 0.17 ± 0.08 × 10^5 ^RU/g per L. The Spearman's rho analysis showed that there was a highly positive association between the expressions of GRP78 of plasma cells from synovial fluid and the corrected synovial fluid levels of anti-CCP (IgG) in inflamed RA peripheral joints (Figure 4).

## Discussion

The present work showed that there was an apparently increased expression of GRP78 in infiltrating plasma cells of RA synovium compared with that of OA synovium and that there was a statistically significant difference between the mean expressional levels of GRP78 in CD138^+ ^plasma cells from synovial fluids of RA and those of OA. These data suggest that enhanced activation of the UPR in infiltrating plasma cells occurs in the autoimmune microenvironment of rheumatoid synovitis. Previous observations had demonstrated a lower level of immunoglobulin synthesis of infiltrating plasma cells in OA synovium when compared with those in RA synovium [23,24]. In addition, the highly positive association between the expressions of GRP78 of synovial plasma cells and the corrected synovial fluid levels of anti-CCP (IgG), which could be related to the local antigen-driven secondary antibody responses in rheumatoid synovium [2,4,25], was seen in the present study. Collectively, these findings can suggest that the upregulated expression of GRP78 of plasma cells may be at least partially related to the local production of immunoglobulin in rheumatoid synovium.

In this study, 11 of 24 synovia sampled from the RA patients assumed the appearance of follicular synovitis, and this ratio of rheumatoid synovitis was apparently higher than ratios that were previously described (approximately 30%) [6,26]. Our data are similar to those presented in a recent report [27] and may result in part from the fact that rheumatoid synovium with follicular synovitis appears to be seen more frequently at surgeries and the fact that the presence of lymphoid follicles in rheumatoid synovium may imply a greater risk for joint destruction [1,26]. In addition, the increased expression of GRP78 of plasma cells in synovium and synovial fluid of follicular synovitis compared with that of diffuse synovitis was seen in this study. It can be inferred from the previous reports that the immune microenvironment of follicular synovitis can be linked to the combination of lymphoid microstructures and local releases of proinflammatory cytokines, including tumor necrosis factor-α, interferon-γ, and interleukin-6 and interleukin-1β [6,28,29], and the upregulated production of APRIL (a proliferation-inducing ligand) capable of sustaining B-cell development and plasmablast survival [30,31], by infiltrating myeloid dendritic cells in rheumatoid synovium [32]. These locally produced cytokines might facilitate the cognate interactions between follicular dendritic cells, autoreactive T cells, and B cells within the lymphoid follicles and could stimulate the terminal differentiation of B cells into long-lived and high-affinity antibody-secreting plasma cells [33-35] and thus may enhance activation of the UPR of infiltrating plasma cells in synovium with follicular synovitis.

At present, the role of ectopic lymphoid neogenesis in rheumatoid synovium may be unsettled. Some reports have shown potential links between the lymphoid neogenesis and the local production of autoantibodies [5-7], especially anti-CCP (IgG) [36]. But the clinical and biological relevance of synovial lymphoid neogenesis has been questioned recently [37,38]. With respect to the present work, the differential expression of GRP78 of plasma cells in distinct variants of rheumatoid synovitis, and the positive association between the expression of GRP78 of plasma cells and the local production of anti-CCP (IgG) within RA-inflamed peripheral joints, may provide partial evidence for the links between ectopic lymphoid neogenesis in rheumatoid synovium and the differentiation of autoantibody-secreting plasma cells as well as the ongoing local autoimmunity [1,39,40].

## Conclusion

The present work demonstrated the upregulated expression of GRP78 of synovial plasma cells of RA compared with that of OA, suggesting that enhanced activation of the UPR occurs in infiltrating plasma cells within inflamed peripheral joints in RA. In addition, there is a potential link between activation of the UPR of infiltrating plasma cells and the ectopic lymphoid neogenesis as well as the local production of anti-CCP (IgG) in rheumatoid synovium. These findings may have some implications for a better understanding of the pathogenesis of rheumatoid synovitis and the potential therapeutic interventions of RA.

## Abbreviations

Anti-CCP: anti-cyclic citrullinated peptide antibodies; BiP: immunoglobulin heavy-chain-binding protein; BSA: bovine serum albumin; EDTA: ethylenediaminetetraacetic acid; ER: endoplasmic reticulum; FACS: fluorescence-activated cell sorting; FITC: fluorescein isothiocyanate; GRP78: 78-kDa glucose-regulated protein; HE: hematoxylin and eosin; OA: osteoarthritis; PBS: phosphate-buffered saline; RA: rheumatoid arthritis; SP: streptavidin/peroxidase; TBST: 1 mL of Tween 20 in 1 L of Tris-buffered saline; UPR: unfolded protein response.

## Competing interests

The authors declare that they have no competing interests.

## Authors' contributions

WD participated in the design of the study and the isolation of synovial fluid plasma cells, performed the immunohistochemical and double-immunofluorescent staining, Western blotting, and statistical analyses, and drafted the manuscript. XL and CF performed the flow cytometry assay. YF carried out the isolation of synovial fluid plasma cells. ZC and PZ designed the study, organized the investigational works, and reviewed the manuscript. All authors read and approved the final manuscript.
